# Carbohydrate antigen-125, calcium, and hemoglobin as predictive clinical indicator for ocular metastasis in male liver cancer patients

**DOI:** 10.1042/BSR20194405

**Published:** 2020-02-24

**Authors:** Qian-Hui Xu, Pei-Wen Zhu, Biao Li, Wen-Qing Shi, Qi Lin, You-Lan Min, Qian-Min Ge, Qing Yuan, Yi Shao

**Affiliations:** Department of Ophthalmology, The First Affiliated Hospital of Nanchang University, Nanchang 330006, Jiangxi Province, China

**Keywords:** biomarker, CA-125. clinical indicators, liver cancer, ocular metastasis

## Abstract

**Background** Primary liver cancer (PLC) is a common type of cancer among men worldwide. Little is known regarding the relationship of liver cancer with ocular metastasis (OM). Drinking has been also reported to be related not only to the occurrence of liver cancer but also to the causes of some ocular lesions.

**Purpose** A diagnostic standard for the levels of serum biomarkers associated with OM derived from liver cancer in men is urgently needed.

**Material and methods** We examined the association between OM in liver cancer and its serum biomarkers. A total of 1254 male patients with liver cancer were recruited in this retrospective study between July 2002 and December 2012. We assessed the relationship between drinking preference and OM in male patients with liver cancer, and aimed to identify an independent prognostic factor or establish a quantitative indicator for OM.

**Results** By assessing the potential indicators, carbohydrate antigen-125 (CA-125), calcium, and hemoglobin (Hb) were found to be most valuable in the diagnosis of OM in male patients with liver cancer.

**Conclusion** CA-125, calcium, and Hb are independent risk factors of OM in patients with liver cancer who consume alcohol.

## Introduction

Primary liver cancer (PLC) is a common type of malignant tumor in the liver and includes hepatocellular carcinoma (HCC), the incidence of which is the fourth largest worldwide [1]. The mortality rate of liver cancer ranks second in China, at the same time, the disease onset is obvious and the disease progresses rapidly. Therefore, early diagnosis is essential for improving the prognosis [2]. The number of HCC deaths per year is almost the same as the number of cases, highlighting the high mortality rate associated with this invasive disease [3]. The high incidence of HCC is partly related to alcohol consumption [4]. Studies have shown that HCC develops through the long-term interaction of multiple pathways, steps, and factors. The risk factors for liver cancer are mainly hepatitis B virus (HBV) and hepatitis C virus, followed by cirrhosis, aflatoxin, alcoholism, and smoking. Genetic and environmental factors may also increase the risk of liver cancer. At present, studies have identified that HBVx genotype B can promote the migration, invasion, and adhesion of liver cancer cells during the development of liver cancer [5]. In Western countries, cirrhosis is one of the risk factors for the development of HCC. Such changes are rarely observed in non-cirrhotic livers, and persistent drinking is the main cause of cirrhosis [6]. The clinical results obtained in the treatment of patients with HCC are not particularly good whether they are treated with intervention or surgery [7]. A total of 5–15% of patients with alcoholic cirrhosis (AC) may develop HCC, and the incidence rate of HCC among those patients is approximately 1.0–1.5% per year, which is close to the threshold for HCC monitoring (1.5%) [8]. The 5-year cumulative risk is approximately 8%. AC is the cause of 32–45% of liver cancer cases in China [9], and the prognosis of patients is poor because HCC is often detected only at an advanced stage. Reactive oxygen species (ROS) is produced during alcohol metabolism, and if it is not eliminated completely, it may cause oxidative stress, which will further lead to inflammatory response of Kupffer cells and lipid peroxidation [10], thus long-term past may lead to the occurrence of liver cancer. According to this evidence, alcohol consumption is considered a risk factor for liver cancer in men. In addition, clinical research identified that alcohol consumption can also cause eye disease, as the methanol contained in alcohol exerts obvious toxic side effects on the retina. We suspect that alcohol consumption may be associated with ocular metastasis (OM) in patients with liver cancer due to two pathogenic effects. However, in the present study, we were unable to draw accurate conclusions from the relatively small number of samples. Nevertheless, the results showed an important relationship between alcohol and PLC. PLC is often accompanied by lung metastasis, abdominal metastasis, mediastinal lymph node metastasis, and even brain metastasis [11–13]. The eye is a rare site for liver cancer metastasis owing to the limited number of blood vessels [14] and lymphatic vessels [15] in this organ. Currently, clinical screening methods for early liver cancer mainly include the detection of serum α fetoprotein (AFP) and ultrasound examination of the liver. However, the detection of AFP sensitivity and specificity is limited and has a high rate of missed diagnosis [16]. Moreover, ultrasound examination largely relies on the subjective judgment of the operator, and it is difficult to effectively identify liver-occupying lesions through conventional ultrasound. Therefore, it is essential to develop a more effective and accurate screening method for liver cancer. Tumor markers have great significance in improving the early detection of liver cancer, as well as the prognosis, survival rate, and quality of life of patients. An increasing number of liver cancer biomarkers with high sensitivity and specificity, such as AFP isoform 3 (AFP-L3), have been identified and routinely applied in clinical testing [17]. Tumor markers are molecules that indicate the presence of tumor cells. These markers can appear in the blood of patients with tumors, and different types of tumors can have different types of markers. Targeted detection of related tumor markers in the serum may promote the early detection and timely treatment of malignant tumors. Tumor markers are produced by tumors and accumulate in other tissues and body fluids [18]. Effective blood tests have prognostic value and can help clinicians make treatment decisions. Therefore, serum biomarkers have been studied for a long time to establish reliable criteria for diagnosing metastases, which can be combined with computed tomography (CT) scans and tissue biopsies. In our retrospective analysis, potential serum indicators associated with OM in male HCC patients who consumed alcohol were analyzed, and diagnostic criteria were established to distinguish OM from non-OM (NOM) in male patients with HCC and achieve the predictive diagnosis and early treatments of male liver cancer patients with OM.

## Materials and methods

### Study design

The present study was approved by the Medical Research Ethics Committee of the First Affiliated Hospital of Nanchang University, Nanchang, Jiang, China and conducted in strict accordance with the tenets of the Declaration of Helsinki. The methods used in the present study were all in accordance with the standards that all the patients signed the consent form, and the clinical trials have been registered as legislation requires. From July 2002 to December 2012, serum samples from male patients with liver cancer were collected preoperatively. We observed a total of 1254 male patients with liver cancer admitted to First Affiliated Hospital of Nanchang University, Nanchang, Jiang, China. Based on the histopathological examination of biopsy samples obtained by surgical resection or needle biopsy, the patients were divided into two groups: OM group and NOM group. Diagnosis of OM was determined by CT or magnetic resonance imaging (MRI), and confirmed through histology or cytology. All subjects provided written informed consent prior to their participation in this clinical study.

### Measurement of tumor markers

Five milliliters of fasting venous blood from the male patients with liver cancer were taken and centrifuged at 3000 rpm for 10 min, then serum was taken and stored at −20°C, and detection took place within 6 h. The detection of serum tumor marker levels was by chemiluminescence immunoassay and ELISA.

### Data collection

All male patients with liver cancer confirmed by CT and MRI were recruited in the study. We collected clinical data, including age, alcohol consumption, and pathological type, from the clinical medical records of these patients. Additionally, we analyzed the levels of several tumor markers including blood calcium concentration, hemoglobin (Hb), alkaline phosphatase (ALP), ferritin (FER), carcinoembryonic antigen (CEA), carbohydrate antigen-125 (CA-125), carbohydrate antigen-153 (CA-153), carbohydrate antigen-199 (CA-199), carbohydrate antigen-724 (CA-724), serum lipids (e.g., total cholesterol (TC), triglycerides, high-density lipoprotein (HDL) cholesterol, and low-density lipoprotein (LDL) cholesterol), lipoprotein(a) (Lp(a)), apolipoprotein A-I (ApoA1), and apolipoprotein B (ApoB), from patients’ serum.

### Statistical analyses

Student’s *t* test and the chi-squared test were used to compare the differences between the OM and NOM groups. The significance of serum biomarkers in eye metastasis in male patients with liver cancer was assessed using a binary logistic regression model. The area under the curve (AUC), sensitivity and specificity were calculated by generating receiver operating characteristic (ROC) curves to reach the diagnosis. A *P*<0.05 in the bilateral test denoted a statistically significant difference. Statistical analyses were conducted using the SPSS version 21.0 software (SPSS Inc., IBM Corp., Armonk, NY, U.S.A.), MedCalc18.6.0 statistical software (MedCalc, Ostend, Belgium), and Excel 2016 software (Excel, Microsoft Corporation, Redmond, WA, U.S.A.). Clinical measurement data were expressed as mean ± standard deviation (SD).

## Results

### Demographics and characteristics of patients

**Figure 1:**
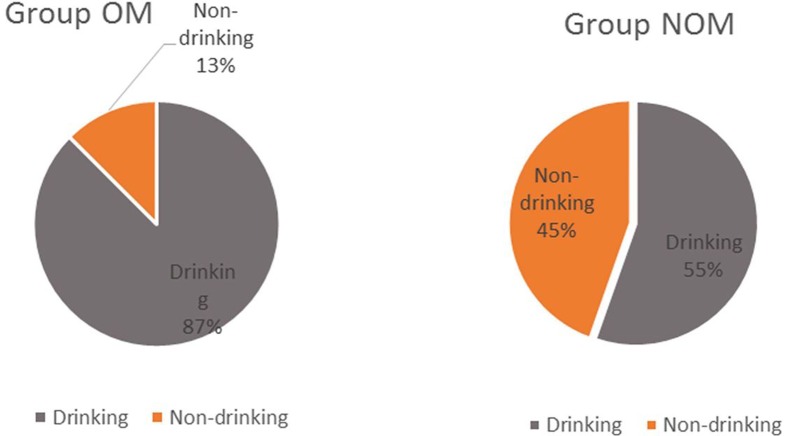
The proportion of drinkers and non-drinkers in the OM and NOM groups

A total of 1254 male patients with liver cancer were enrolled in the study, including 16 OM patients and 1238 NOM patients. The mean age in the OM and NOM groups was 54.0 ± 3.0 and 52 ± 1.4 years, respectively. The chi-squared and non-parametric summary tests did not reveal significant differences in age between the OM group and NOM group (*P*>0.05), while a significant difference was observed between the groups in drinking preference (*P*<0.05), which confirms that drinking is related to the occurrence of liver cancer metastasis in males. The clinical manifestations and data for all patients participating in the study are shown in Table 1. Table 2 shows the number of drinkers and non-drinkers in the OM and NOM groups. Figure 1 shows the percentage of drinkers and non-drinkers in the OM and NOM groups.

**Table 1:** Characteristics of male patients with liver cancer who consumed alcohol

Patient characteristics	OM group^1^ (%) (*n*=16)	NOM group (%) (*n*=1238)	*P*-value^4^
**Age**^3^			*P*>0.05
Mean	54.0 ± 3.0	52 ± 1.4	
**Histopathological type**^2^			0.624
HCC	9 (0.56)	590 (0.48)	
Intrahepatic cholangiocarcinoma (ICC)	3 (0.19)	147 (0.12)	
Other	4 (0.25)	487 (0.40)	

*P*<0.05 indicates statistical significance.

^1^OM, including intraocular metastasis and eyelid metastasis.

^2^Chi-squared test.

^3^Student’s *t* test.

^4^Comparison between the OM and NOM groups.

**Table 2:** The histopathological types of liver cancer in male patients who consumed and did not consume alcohol

OM^1^	OM (%) (*n*=16)	NOM (%) (*n*=1238)	*P*-value^2^
Drinking	14 (0.875)	682 (0.551)	0.011
Non-drinking	2 (0.125)	556 (0.449)	

*P*<0.05 indicates statistical significance.

^1^Chi-squared test was used.

^2^Comparison between the OM and NOM groups.

### Differences in the clinical manifestations, the level of tumor markers, and risk factors of OM

The concentrations of CA-125, CA-724, LDL, and calcium in the serum of patients in the OM group were significantly higher than those observed in the NOM group (*P*<0.05). In contrast, the Hb levels were significantly lower than those reported in the NOM group (*P*<0.05). The differences in the levels of CA-199, CA-153, CEA, FER, ALP, ApoA1, ApoB, TC, HDL, and Lp(a) between the two groups were not significant (*P*>0.05). The detailed results are shown in Table 3. Binary logistic regression analysis showed that the levels of CA-125, calcium, and Hb in the serum were independent risk factors of OM. The detailed results are shown in Table 4.

**Table 3:** Differences in the concentration of various tumor biomarkers between male patients with liver cancer who consumed alcohol with and without OM

Tumor biomarkers	OM group	NOM group	*t* test	*P*-value
CEA (ng/ml)	13.85 ± 29.59	6.48 ± 36.41	−0.806	0.228
CA-125 (U/ml)	405.62 ± 342.64	125.76 ± 253.99	−3.256	0.012
CA-199 (U/ml)	103.08 ± 211.97	107.37 ± 722.51	0.023	0.864
CA-153 (U/ml)	19.67 ± 14.42	19.95 ± 22.16	0.049	0.863
CA-724 (U/ml)	7.69 ± 10.08	6.88 ± 6.28	−0.320	0.021
FER (U/l)	258.61 ± 272.59	253.17 ± 190.85	−0.113	0.847
ALP (U/l)	177.19 ± 81.92	164.64 ± 152.27	−0.329	0.361
TC (mmol/l)	4.85 ± 1.91	5.63 ± 10.98	0.285	0.536
TG (mmol/l)	1.59 ± 1.39	1.36 ± 0.99	−0.891	0.255
HDL (mmol/l)	1.70 ± 1.35	1.45 ± 0.99	−1.007	0.279
LDL (mmol/l)	3.10 ± 2.05	2.50 ± 1.28	−1.170	0.032
ApoA1 (g/l)	1.67 ± 0.47	1.53 ± 0.44	−1.313	0.337
ApoB (g/l)	0.98 ± 0.45	1.05 ± 0.75	0.404	0.259
Lipoprotein-a (mg/l)	181.50 ± 216.39	221.16 ± 238.86	0.661	0.745
Calcium (mmol/l)	12.27 ± 40.46	2.29 ± 4.61	−0.987	<0.001
Hb (g/l)	118.13 ± 38.90	120.38 ± 23.57	0.231	0.044

Independent sample *t* test. *P*<0.05 denotes statistical significance. Abbreviation: TG, triglyceride.

**Table 4:** Risk factors for OM in male patients with liver cancer who consumed alcohol

Factors	B	Exp (B)	OR (95% CI)	*P*-value
CEA (ng/ml)	0.004	0.995–1.012	1.004	0.423
CA-125 (U/ml)	0.005	1.003–1.007	1.005	<0.001
CA-199 (U/ml)	0.000	0.998–1.001	1.000	0.804
CA-153 (U/ml)	−0.011	0.959–1.020	0.989	0.482
CA-724 (U/ml)	0.024	0.950–1.104	1.024	0.530
FER (U/l)	0.000	0.998–1.003	1.000	0.867
ALP (U/l)	0.000	0.994–1.005	1.000	0.852
TC (mmol/l)	−0.136	0.514–1.481	0.873	0.614
TG (mmol/l)	−0.184	0.381–1.817	0.832	0.645
HDL (mmol/l)	−0.107	0.391–2.067	0.899	0.802
LDL (mmol/l)	0.397	0.749–2.950	1.487	0.257
Apolipoprotein A (g/l)	0.900	0.654–9.239	2.459	0.183
ApoB (g/l)	−0.691	0.158–1.588	0.501	0.240
Lipoprotein-a (mg/l)	−0.001	0.996–1.002	0.999	0.558
Calcium (mmol/l)	0.060	1.028–1.096	1.062	<0.001
HB (g/l)	0.031	1.003–1.003	1.031	0.032

Binary logistic regression analysis. *P*<0.05 denotes statistical significance. Abbreviations: B, coefficient of regression; CI, confidence interval; OR, odds ratio; TG, triglyceride.

### The cut-off value, AUC, sensitivity, and specificity of CEA and LDL-C for the diagnosis of OM

**Figure 2:**
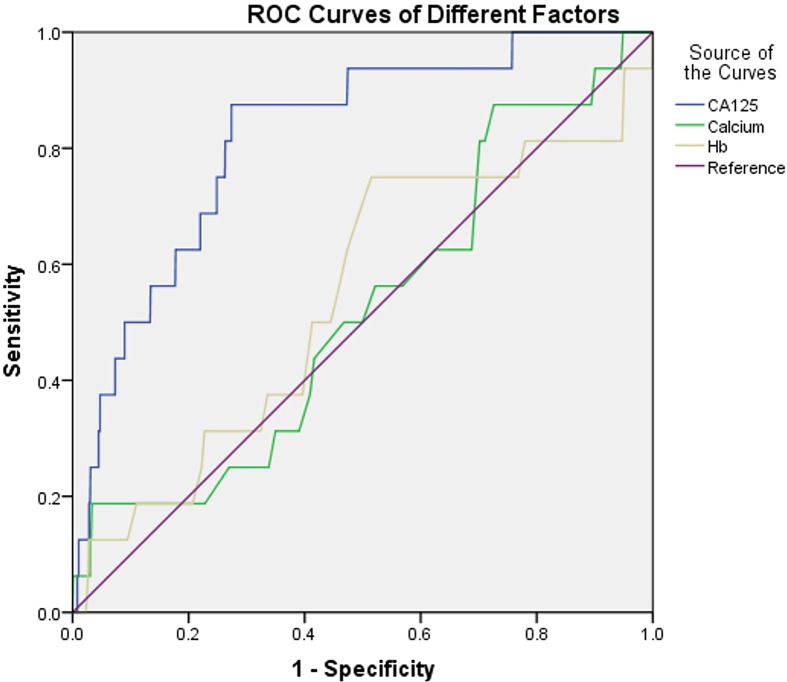
ROC curves of different factors The ROC curves of risk factor for detecting OM in metastatic liver cancer. ROC curves of CA-125, calcium and Hb as single risk factor of OM. Abbreviation: CA, carbohydrate antigen.

Figure 2 shows the ROC curves for the level of CA-125, calcium, and Hb. Table 5 shows the cut-off values, which were 115.13 U/ml, 2.65 mmol/l, and 120.50 g/l, respectively. The AUC of CA-125 was the highest for any single factor, reaching 0.82. Among all the possible indicators, CA-125 exhibited the highest sensitivity and specificity for blood calcium. All results were statistically significant.

**Table 5:** The cut-off values, sensitivity, specificity, and AUC of risk factors for the prediction of OM in male patients with liver cancer who consumed alcohol

Factor	Cut-off value	Sensitivity (%)	Specificity (%)	AUC	*P*-value
CA-125	115.13	87.50	72.50	0.820	<0.001
Calcium	2.65	18.80	96.70	0.521	<0.001
Hb	120.50	75.00	48.50	0.543	0.032

Sensitivity and specificity were determined at the cut-off value. *P*<0.05 denotes statistical significance.

## Discussion

Liver cancer, the fourth most common malignant tumor observed in clinical practice, is characterized by high malignancy, rapid progression, high invasiveness, poor prognosis, and high mortality, lowering the quality of life of patients [3]. HCC is one of the PLCs with a high prevalence [1]. Studies have shown that liver cancer metastasizes through the blood. A total of 75% of liver cancer metastasis is the lung metastasis, besides chest and lumbar vertebrae metastases that account for 11% of metastases. If liver cancer metastasizes to the brain, patients often experience severe nausea and headaches. The mortality rate associated with PLC is 54% worldwide [4], and accurate diagnosis at an early clinical stage is thought to greatly improve treatment efficacy and patient survival. However, after surgery, the high rates of metastasis and recurrence of PLC (mainly HCC) are the main factors affecting prognosis [19]. Therefore, an in-depth study of OM in HCC is essential. Metastasis of liver tumors to the eye has not been reported in detail in the current research.

However, the treatment of this type of cancer is very challenging. With the improvements in tumor diagnosis and treatment, including liver surgery, an increasing number of clinicians need to implement this technology in their practice. Although there are many treatment modalities against metastatic liver cancer, the prognosis of this disease is currently not satisfactory. Wayne et al. [20] found that tumor size and portal vein tumor thrombus were the main factors associated with recurrence after surgery for liver cancer. Studies have shown that men are more likely to develop liver cancer than women.

The American Cancer Society recommends that limiting alcohol consumption and practicing good lifestyle habits can prevent 25–30% of cancer cases [21], even though these recommendations are controversial. In a study performed by Scoccianti et al. [22], alcohol consumption was found to be related to male cancers. According to some studies, drinking may lead to the occurrence of cancer [25]. Thus, based on the findings reported by Scoccianti et al. [23], tumor markers may be potentially useful in predicting tumor metastasis. Studies have reported that Apos are strongly correlated with the development of cancer, for example, breast cancer patients with OM may have lower levels of ApoA1 compared with NOM patients. Animal experiments have shown that specific Apos may exert an effect on tumor growth by modulating the function of immune cells [24]. The levels of other Apos can be used to determine the prognosis of patients with different cancers [25,26]. In previous studies, it was shown that the occurrence of liver cancer metastasis is related to different serum tumor markers, intracranial metastasis is the most common site of central nervous system metastasis of liver cancer. ALP can be used as a tumor biomarker to predict central nervous system metastasis of liver cancer [27]. Studies also have shown the clinical value of tumor markers in predicting metastasis of different types of tumors, serum tumor markers HDL, TC, and ApolB are risk factors for colorectal cancer [28].

We investigated the association between OM and liver cancer. First, we collected serum from patients with liver cancer and analyzed the concentration of several kinds of tumor markers. After obtaining serum from a large number of patients, we measured the concentration of CA-125, calcium, and Hb as independent risk factors of OM in patients with male liver cancer who consume alcohol (*P*<0.05). Second, we determined the cut-off values, sensitivity, and specificity. Finally, we concluded that CA-125, calcium, and Hb are important risk factors of OM in patients with liver cancer. Using the ROC curve, we found that the levels of CA-125 >115.13 U/ml, calcium >2.65 g/l, and Hb >120.50 g/l indicate an increased risk of OM among male liver cancer patients who consume alcohol, all of them have the statistical significance, which can be used for clinical application. It is obvious that based on detailed diagnostic techniques (e.g., CT and MRI of the eye), the AUC of CA-125 yielded the highest value. CA-125 has high accuracy for the diagnosis of the OM of liver cancer.

In the present study, we also assessed the relationship between drinking and ocular diseases. Alcohol exerts a direct effect on the retina, preventing the retina from producing a sensory visual pigment. Excessive drinking can cause digestive and absorption dysfunction, leading to vitamin deficiency, which can cause conjunctivitis, optic neuritis, and other eye diseases, especially in patients with diabetes and high blood pressure. When we compared the differences between OM and NOM patients from Table 2 and Figure 1, we found that male patients with liver cancer who consume alcohol are more likely to develop ocular metastases.

The present research has some limitations, and further research is needed. Studies with larger sample sizes conducted at multiple centers would improve the effectiveness of these risk factors in predicting OM in male patients with liver cancer who consume alcohol. The metastatic stage of liver cancer is usually fatal. Besides, the sample has the limitation of districts which only studied the male liver patients in Nanchang.

## Conclusion

CA-125 shows higher predictive value among various risk factors of OM in liver cancer owing to its high sensitivity, specificity, and AUC. Drinking is also a potential risk factor of OM in male patients with liver cancer. Multicenter prospective studies are warranted to verify the present results and improve the screening, diagnosis, and treatment of the affected population.

## Data Availability

The datasets generated during and/or analyzed during the current study are available from the corresponding author on reasonable request.

## Abbreviations

AC: alcoholic cirrhosis
AFP: α fetoprotein
AFP-L3: α fetoprotein isoform 3
ALP: alkaline phosphatase
ApoA1: apolipoprotein A-I
ApoB: apolipoprotein B
Apos: apolipoproteins
AUC: area under the curve
CA-125: carbohydrate antigen-125
CA-153: carbohydrate antigen-153
CA-199: carbohydrate antigen-199
CA-724: carbohydrate antigen-724
CEA: carcinoembryonic antigen
CT: computed tomography
FER: ferritin
HBV: hepatitis B virus
Hb: hemoglobin
HCC: hepatocellular carcinoma
HDL: high-density lipoprotein
ICC: intrahepatic cholangiocarcinoma
LDL: low-density lipoprotein
Lp(a): lipoprotein(a)
MRI: magnetic resonance imaging
NOM: non-ocular metastasis
OM: ocular metastasis
PLC: primary liver cancer
ROC: receiver operating characteristic
TC: total cholesterol
